# Salicylic acid amplifies Carbachol-induced bronchoconstriction in human precision-cut lung slices

**DOI:** 10.1186/s12931-019-1034-x

**Published:** 2019-04-11

**Authors:** Joseph Jude, Danielle Botelho, Nikhil Karmacharya, Gao Yuan Cao, William Jester, Reynold A. Panettieri

**Affiliations:** 1 Rutgers Institute for Translational Medicine and Science (RITMS), Rutgers, The State University of New Jersey, Rm# 4276, 89 French Street, New Brunswick, NJ08901 USA; 2 Research Institute for Fragrance Materials (RIFM), Woodcliff Lake, New Jersey USA

## Abstract

**Background:**

Asthma exacerbations evoke emergency room visits, progressive loss of lung function and increased mortality. Environmental and industrial toxicants exacerbate asthma, although the underlying mechanisms are unknown. We assessed whether 3 distinct toxicants, salicylic acid (SA), toluene diisocyanate (TDI), and 1-chloro-2,4-dinitrobenzene (DNCB) induced airway hyperresponsiveness (AHR) through modulating excitation-contraction coupling in human airway smooth muscle (HASM) cells. The toxicants include a non-sensitizing irritant (SA), respiratory sensitizer (TDI) and dermal sensitizer (DNCB), respectively. We hypothesized that these toxicants induce AHR by modulating excitation-contraction (EC) coupling in airway smooth muscle (ASM) cells.

**Methods:**

Carbachol-induced bronchoconstriction was measured in precision-cut human lung slices (hPCLS) following exposure to SA, TDI, DNCB or vehicle. Culture supernatants of hPCLS were screened for mediator release. In HASM cells treated with the toxicants, surrogate readouts of EC coupling were measured by phosphorylated myosin light chain (pMLC) and agonist-induced Ca^2+^ mobilization ([Ca^2+^]_i_). In addition, Nrf-2-dependent antioxidant response was determined by NAD(P) H quinone oxidoreductase 1 (NQO1) expression in HASM cells.

**Results:**

In hPCLS, SA, but not TDI or DNCB, potentiated carbachol-induced bronchoconstriction. The toxicants had little effect on release of inflammatory mediators, including IL-6, IL-8 and eotaxin from hPCLS. In HASM cells, TDI amplified carbachol-induced MLC phosphorylation. The toxicants also had little effect on agonist-induced [Ca^2+^]_i._

**Conclusion:**

SA, a non-sensitizing irritant, amplifies agonist-induced bronchoconstriction in hPCLS via mechanisms independent of inflammation and Ca^2+^ homeostasis in HASM cells. The sensitizers TDI and DNCB, had little effect on bronchoconstriction or inflammatory mediator release in hPCLS.

**Implications:**

Our findings suggest that non-sensitizing irritant salicylic acid may evoke AHR and exacerbate symptoms in susceptible individuals or in those with underlying lung disease.

**Electronic supplementary material:**

The online version of this article (10.1186/s12931-019-1034-x) contains supplementary material, which is available to authorized users.

## Background

Asthma exacerbations evoke emergency room visits, deterioration in lung function and even mortality. Chemical toxicants can trigger asthma exacerbations. The underlying mechanisms of toxicant-associated exacerbations remain unknown. Inflammation, oxidative injury and immune sensitization are putative mechanisms through which toxicants elicit AHR, although compelling evidence suggest that some toxicants induce AHR uncoupled from inflammation [1]. ASM is a pivotal tissue regulating bronchomotor tone and a primary modulator of bronchoconstriction in asthma and COPD.

To assess whether ASM function is differentially modulated by toxicants, we chose to study the effects of a non-sensitizing irritant, a respiratory or a dermal sensitizer on excitation-contraction (EC) coupling in human ASM (HASM) cells. The non-sensitizing irritant salicylic acid (SA) is a plant extract used as an exfoliating agent in cosmetic products. Irritant-induced asthma is a category of occupational asthma characterized by lack of sensitization or adaptive immune response [2–4]. Sensitizers rely on innate and adaptive immune responses with antibody production to elicit their adverse reaction. Toluene diisocyanate (TDI), a manufacturing intermediate in many synthetic materials, is a respiratory sensitizer and a commonly reported cause of occupational asthma [5, 6]. Since 1970s, there is a decline in TDI-induced occupational asthma incidence rates, largely due to engineering controls at work places to minimize exposure [7]. However, studies seeking to determine exposure-response relationship found that cumulative exposure to lower levels of TDI can accelerate decline in lung function (measured by forced expiratory volume in 1 s -FEV_1_, [8–10]. Dinitrochlorobenzene (DNCB) and its chemical variants are dermal sensitizers. Innate and adaptive immune cellular responses are implicated in DNCB-induced contact hypersensitivity (reviewed in [11]). In animal models, inhaled DNCB reportedly elicit allergic inflammation and sensitization with little effect on breathing mechanics [12–15].

ASM cells play a pivotal role in AHR through their synthetic, mechanical and remodeling functions [16, 17]. In ASM cells, agonist-induced mobilization of Ca^2+^ from intracellular stores elevates cytosolic Ca^2+^ ([Ca^2+^]_i_), which in turn binds to calmodulin and activates myosin light chain (MLC) kinase [18, 19]. MLC kinase phosphorylates MLC and induces actomyosin cross-bridge cycling and ASM cell shortening (reviewed in [20]). Alternatively, Ca^2+^ sensitization mechanism inhibits MLC phosphatase activity and sustains elevated p-MLC level to increase actomyosin cross-bridge cycling [21]. Therefore, agonist-induced [Ca^2+^]_i_ and MLC phosphorylation levels are considered the surrogate measures of EC coupling in ASM cells. Others and we have reported that toxicants can modulate these signaling pathways to increase bronchomotor tone or elicit AHR [1, 22–24]. Recent evidence also suggests that actin polymerization and cytoskeletal reorganization could elicit ASM cell shortening independent of cellular Ca^2+^ homeostasis and MLC phosphorylation [25].

In this study, using precision-cut human lung slices (hPCLS) and primary human airway smooth muscle (HASM) cells, we tested the central hypothesis that the toxicants SA, TDI and DNCB induce AHR by modulating EC coupling in ASM cells. Our findings show that the non-sensitizing irritant SA induces AHR independent of inflammatory mediator release, while TDI or DNCB has little effect on AHR or inflammatory mediator release. The sensitizer TDI increased agonist-induced MLC phosphorylation in HASM cells, indicating modulation of EC coupling in HASM cells.

## Materials and methods

### Reagents

HAM’s F-12 medium, PBS, FBS, 0.05% Trypsin and EDTA, PAGE/western blotting supplies and Lipofectamine RNAimax were purchased from Life Technologies (Grand Island, NY). Antibodies for pMLC (p^S18/T19^-MLC), total MLC and tubulin were purchased from Cell Signaling Technology (Danvers, MA). Antibody for NQO1 were purchased from Santa Cruz Biotechnology (Santa Cruz, CA). Duoset ELISA kits for IL-6, IL-8 and eotaxin were obtained from R&D Biosystems (Minneapolis, MN). SiRNA were purchased from Dharmacon (Lafayatte, CO). Fluo-8 calcium flux assay kit was purchased from abcam (Cambridge, MA). LDH cytotoxicity assay kit was purchased from Thermo scientific (Rockford, IL). All other reagents, including the 3 toxicants, were purchased from Sigma Aldrich (St.Loius, MO).

### Culture of HASM cells

Primary HASM cells were harvested, characterized and grown in culture as described in our previous publications [26]. Cells were used in experiments within the first 4 passages to ensure proper smooth muscle phenotype. HASM cells were serum-deprived for 48 h prior to experimental exposures.

### Human precision-cut lung slices (hPCLS) and carbachol concentration-response

Normal human lungs were obtained through National Disease Research Interchange (NDRI, Philadelphia, PA) or International Institute for the Advancement of Medicine (IIAM, Edison, NJ). Samples were de-identified and therefore exempted by the Rutgers University Institutional Review Board. PCLS were prepared as previously described [27, 28]. Carbachol concentration-response experiments were conducted by exposing hPCLS containing small airways to incremental concentrations of carbachol (10^− 8^–10^− 4^ M) for 10 min each and capturing airway luminal narrowing by a supravital microscope. Airway luminal area for each CCh concentration was expressed as the percentage of baseline luminal area. Semi-logarithmic concentration response curves were used to determine agonist concentration of half-maximal response (Log EC_50_), maximal response (E_max_) and area under the curve (AUC) as the pharmacological parameters.

### Exposure to toxicants

Human PCLS or HASM cells were exposed to toxicants in F-12 culture medium. Salicylic acid (SA) was dissolved in serum-free F-12 medium. TDI and DNCB stock solutions were prepared in 200-proof ethanol. All subsequent dilutions were made in serum-free F-12 medium. The following concentrations of the toxicants were used in hPCLS: SA, 0.1, 1 & 10 uM; TDI, 0.01, 0.1 & 1 uM; DNCB 0.1, 1 & 10 uM. HPCLS or HASM cells were exposed to toxicants 24 h before determining agonist-induced bronchoconstriction, mediator release, MLC phosphorylation or Ca^2+^ mobilization. HASM cells were exposed to 10 uM carbachol for 10 min to determine MLC phosphorylation. Concentrations of the toxicants were determined partly based on published cytotoxicity data [29]. Viability of hPCLS or HASM cells in the presence of toxicants were assessed by LDH activity level in culture supernatants (Fig. 2d & e). In hPCLS, preliminary experiments were conducted to determine whether each toxicant, in their highest non-toxic concentration, has effect on bronchoconstriction in hPCLS a) upon acute (10 min) exposure, and b) following 24 h exposure. Since acute exposure of each toxicant had little effect on bronchoconstriction, prolonged exposure regime was used for the remainder of the study. In HASM cells, the toxicants were used at 10-fold lesser concentration than what is used in hPCLS.

### Determination of [Ca^2+^]_i_ in HASM cells

Agonist-induced [Ca^2+^]_i_ in HASM cells was determined as previously described [1] with some modifications. Briefly, HASM cells grown to confluence in a 48-well plate were loaded with fluo-8 Ca^2+^-binding dye. Carbachol (10 uM) or histamine (1 μM) were used to elicit Ca^2+^ response in HASM cells. Fluorescence intensity was monitored for up to 2 min following agonist injection. Area under the curve (AUC) of the time-dependent fluorescence (relative fluorescence units- RFU) was calculated from the response curve.

### Mediator release from PCLS

Preliminary screening for inflammatory mediators was performed using a custom-designed Luminex® multi-analyte array to measure 12 cytokines and chemokines listed in Additional file 1: Table S1 (R&D Systems, Minneapolis, MN). The toxicants had little effect on release of those 12 mediators from hPCLS (data not shown). Subsequently, IL-6, IL-8 and eotaxin were measured in hPCLS supernatants using Duo-Set ELISA kits following manufacturer’s instructions. TNF-α (10 ng/ml) and IL-13 (100 ng/ml) were used as the positive controls for induction of IL-6/IL-8 and Eotaxin, respectively. BCA protein assay was used to determine the protein concentration in the supernatants and the analyte quantity was normalized to the total protein in the supernatant. LDH activity in the supernatants were measured following manufacturer’s protocol (Pierce LDH cytotoxicity say kit, Thermo Scientific, Rockford, IL). One % Triton-X 100 was used as the positive control to induce maximal LDH release. In HASM cells, 100 μM ceramide was used as the positive control to induce maximal LDH release.

### Transfection of HASM cells

HASM cells were transiently transfected with 50 nM of non-targeting or Nrf-2-targeting siRNA as previously described [1]. Cells were exposed to DNCB (0.1 and 1 uM) 72 h post-transfection.

### Data analysis

HASM cells or hPCLS from at least 3 donors were used in the experiments (*n* = 3 donors). In hPCLS studies, from each donor, minimum 3 slices per experimental group were used as technical replicates. Data are expressed as mean or mean ± SEM. GraphPad Prism 5.0 was used for statistical analysis and means were considered significantly different when *p* ≤ 0.05.

## Results

### Salicylic acid enhances carbachol-induced bronchoconstriction in hPCLS

**Fig. 1:**
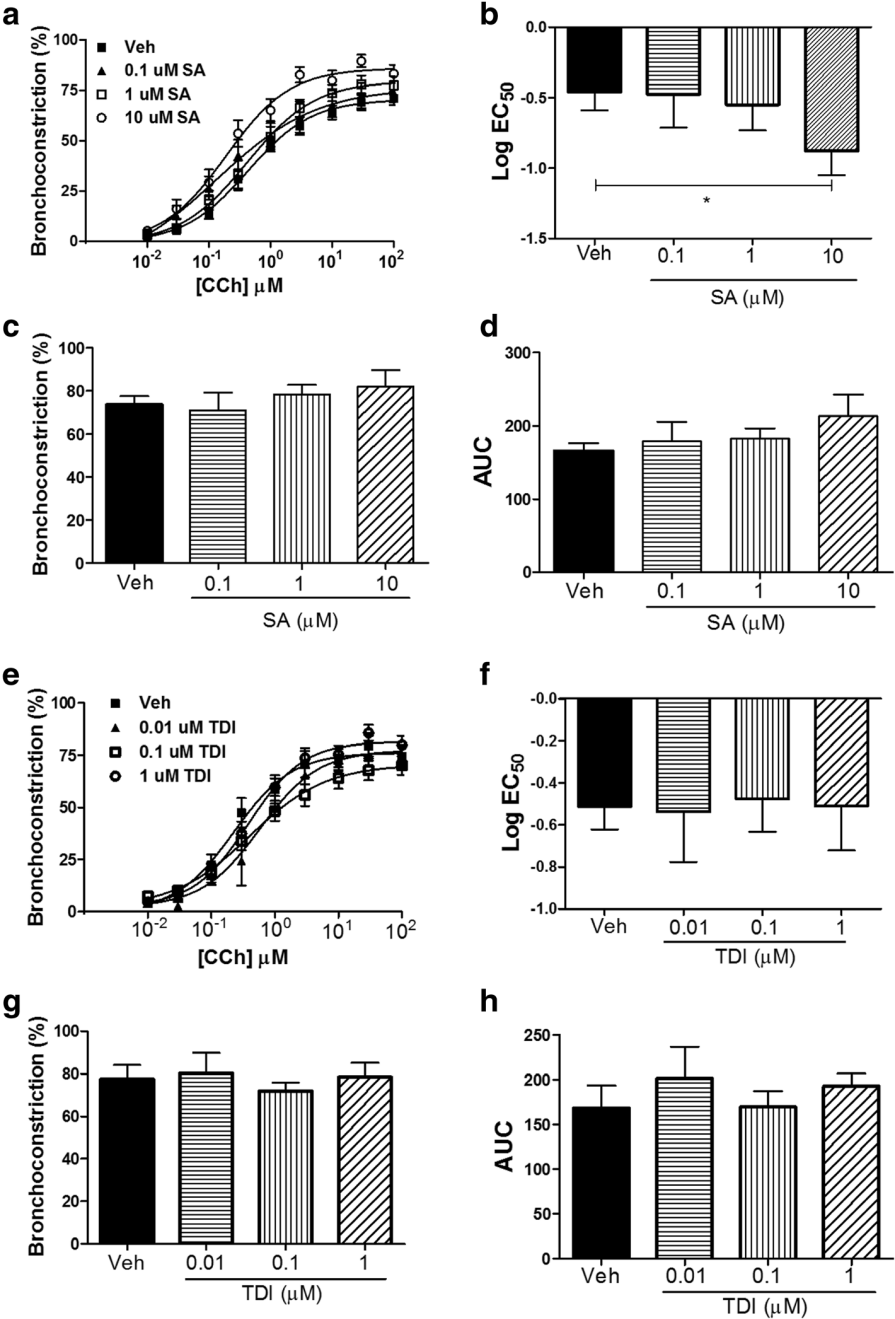
Salicylic acid enhances carbachol-induced bronchoconstriction in hPCLS. Precision-cut human lung slices (hPCLS) from normal human lung donors were exposed to salicylic acid (SA, vehicle F-12 medium) or toluene diisocyanate (TDI, vehicle 0.1% ethanol) for 24 h, followed by a carbachol (CCh) concentration-response for bronchoconstriction. **a** & **b**) SA (10 uM) significantly increased CCh-induced bronchoconstriction (*n* = 4–5 donors, Log EC_50_, *p = 0.04 Veh vs SA 10 uM). **e**-**h**) TDI has no significant effect on CCh-induced bronchoconstriction (*n* = 3–6 donors, Log EC_50_, *p* = 0.4 Veh Vs TDI 1 uM)

To determine whether exposure to toxicants SA, TDI or DNCB potentiated carbachol-induced bronchoconstriction, hPCLS were exposed to vehicle or toxicants for 24 h and carbachol-induced airway narrowing was determined. SA (10 uM) significantly decreased the log EC_50_ (Fig. 1a & b) of the carbachol concentration response curve, suggesting SA enhanced the sensitivity of the airways to the contractile agonist. SA, however, has little effect on the maximal response (E_max_) or area under the curve (AUC) of the concentration-response curve (Fig. 1c & d). Neither TDI nor DNCB exposure had any significant effect on carbachol-induced bronchoconstriction (Fig. 1e-h, Additional file 2: Figure S1).

### Salicylic acid or toluene diisocyanate has little effect on inflammatory mediator release from hPCLS

**Fig. 2:**
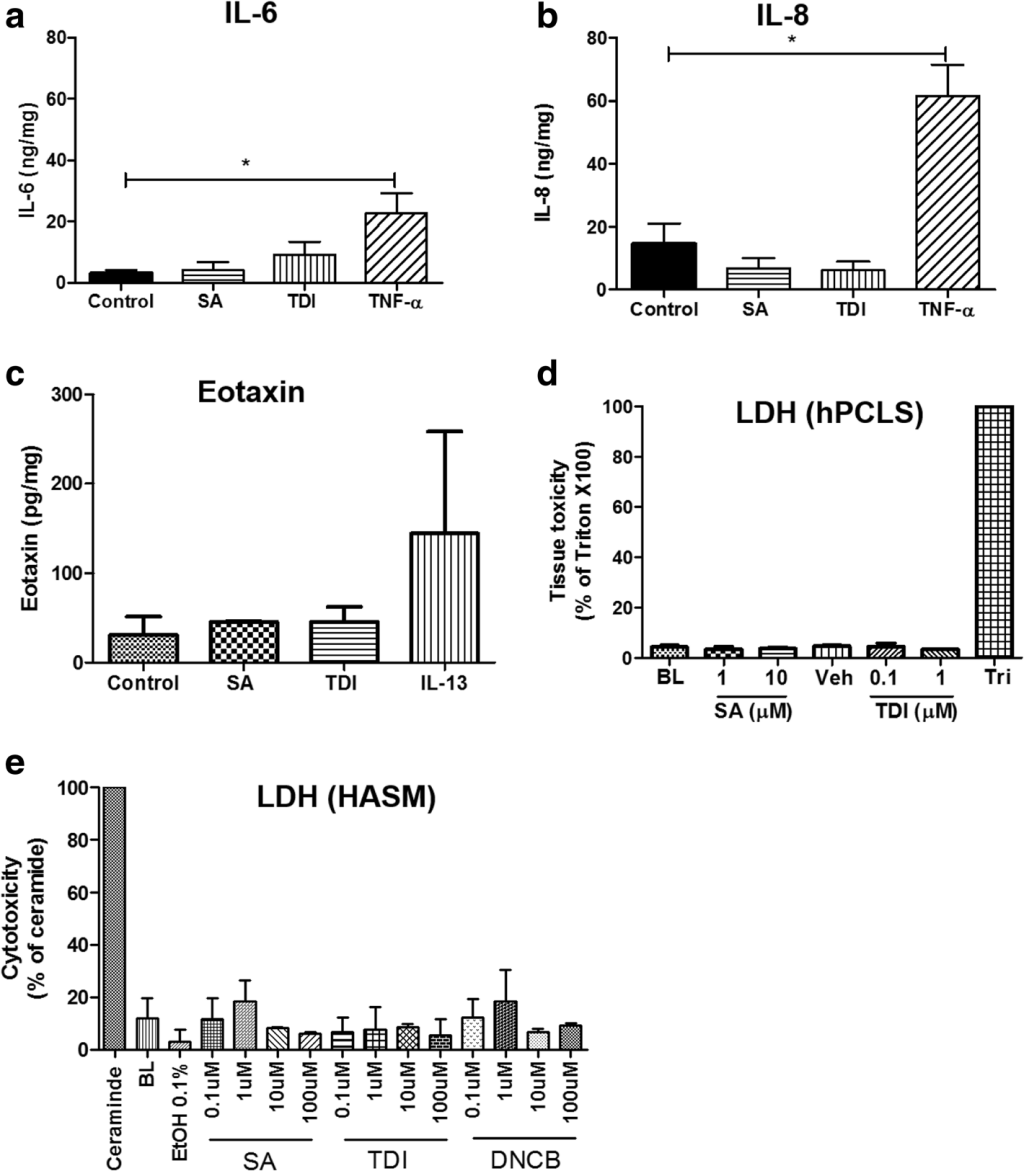
Salicylic acid or toluene diisocyanate has little effect on inflammatory mediator release from hPCLS. Supernatants from human PCLS exposed to salicylic acid (SA 10 uM, vehicle F-12 medium) or toluene diisocyanate (TDI 1 uM, vehicle 0.1% ethanol) for 24 h were screened using a custom Luminex cytokine/chemokine mediator array (Additional file 1: Table S1) to determine mediator release. SA or TDI had no significant effect on any of the 12 mediators analyzed by the array (data not shown). In additional ELISA assays, SA or TDI had little effect on **a**) IL-6, **b**) IL-8 or **c**) Eotaxin release from hPCLS, while the positive controls TNF-α (10 ng/ml) or IL-13 (100 ng/ml) increased IL-6, IL-8 and eotaxin levels, respectively. **d**) SA or TDI has little effect in tissue viability measured by LDH activity in culture supernatant (mean ± SEM of n = 4–5 donors; BL-baseline, Tri-1% Triton X-100). E) The toxicants had < 30% cytotoxicity at 0.1–100 μM (24 h) concentration range in HASM cells, measured by LDH activity in culture supernatants (Mean ± SD of *n* = 2 donors; BL-baseline)

Irritants and sensitizers induce a variety of inflammatory mediators in target tissues to mediate toxicant injury [30]. Since inflammatory mediators are known to induce airway hyper-reactivity [31, 32], we postulated that toxicants SA and TDI may elicit inflammatory mediator release from hPCLS. Lung slices were treated with vehicle, SA or TDI for 24 h and culture supernatants were initially screened for 12 mediators using a custom-designed Luminex® cytokine/chemokine array (Additional file 1: Table S1). SA, TDI or DNCB had little effect on the levels of any of the 12 analytes screened (data not shown). The culture supernatants were further analyzed for their IL-6, IL-8 and eotaxin levels using ELISA. SA (10 uM) or TDI (1 uM) had little effect on IL-6, IL-8 or eotaxin release from hPCLS (Fig. 2 a-c). As a positive control, pro-inflammatory cytokine TNF-α (10 ng/ml), significantly increased release of IL-6 and IL-8 from hPCLS, while IL-13 (100 ng/ml) increased the eotaxin levels. LDH activity assay in the culture supernatants showed that neither SA nor TDI decreased viability of hPCLS (Fig. 2d). The toxicants caused < 30% reduction of viability in HASM cells, when compared to ceramide (Fig. 2e).

### TDI enhances carbachol-induced phosphorylation of myosin light chain in HASM cells

**Fig. 3:**
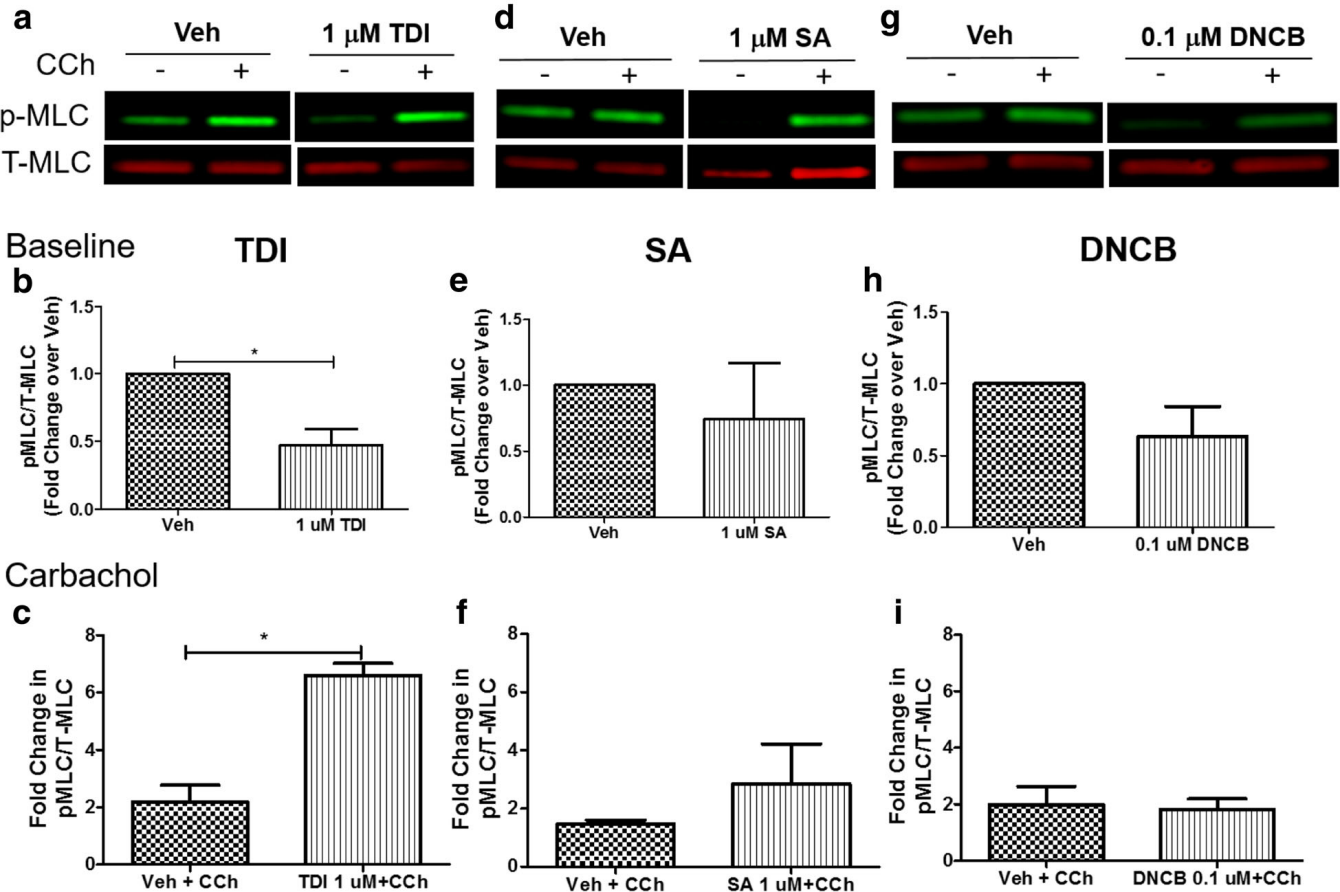
TDI enhances carbachol-induced phosphorylation of myosin light chain in HASM cells. HASM cells were exposed to salicylic acid (SA 1 uM, vehicle F-12 medium) or toluene diisocyanate (TDI 1 uM, vehicle 0.1% ethanol) or dinitrochlorobenzene (DNCB 0.1 uM, vehicle 0.1% ethanol) for 24 h. Baseline or carbachol (CCh)-induced MLC phosphorylation was determined. **a**-**c**) TDI (1 uM) significantly decreased the baseline (*n* = 3 donors, *p = 0.047) MLC phosphorylation, while enhancing carbachol-induced MLC phosphorylation (*n* = 3 donors, *p = 0.03). **d**-**f**) SA or **g**-**i**) DNCB had little effect on baseline or carbachol-induced MLC phosphorylation (n = 3 donors)

Salicylic acid-induced airway hyperresponsiveness (AHR) may be mediated through its direct effect on EC coupling in HASM cells. Our previous studies reported that the indoor air pollutant formaldehyde modulates EC coupling in HASM cells to elicit AHR [1]. To test this possibility, HASM cells were exposed to SA, TDI or DNCB for 24 h and myosin light chain (MLC) phosphorylation was determined in the presence or absence of carbachol. TDI (1 uM) significantly attenuated the baseline level of MLC phosphorylation, while enhancing carbachol-induced MLC phosphorylation (Fig. 3 a-c). SA (Fig. 3 d-f) or DNCB (Fig. 3 g-i) had little effect on baseline or carbachol-induced MLC phosphorylation.

### Salicylic acid or toluene diisocyanate has little effect on agonist-induced [Ca^2+^]_i_ in HASM cells

**Fig. 4:**
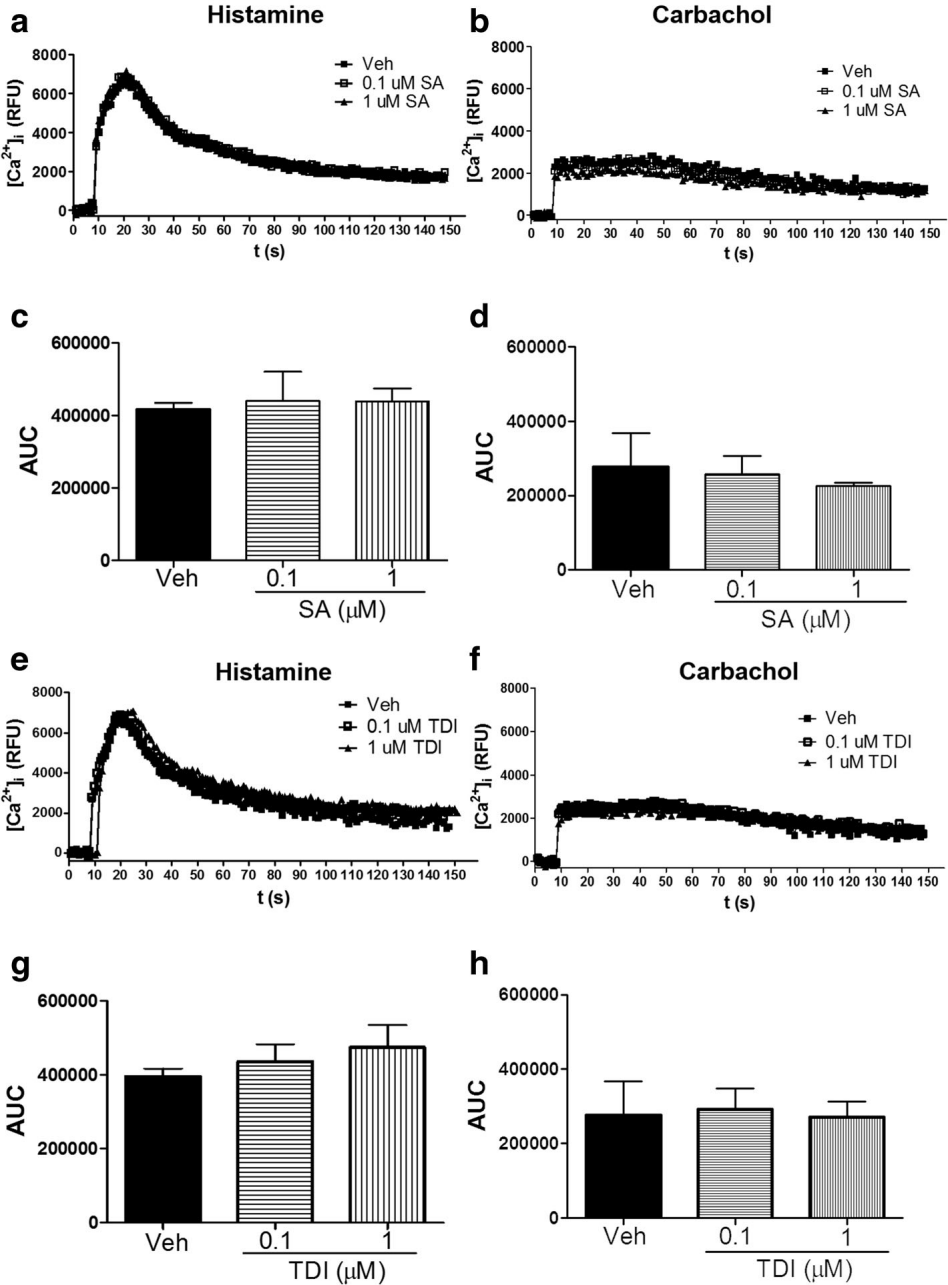
Salicylic acid or toluene diisocyanate has little effect on agonist-induced [Ca^2+^]_i_ in HASM cells. Confluent HASM cells were exposed to salicylic acid (SA: 0.1, 1 uM; vehicle: F-12 medium) or toluene diisocyanate (TDI: 0.1, 1 uM; vehicle: 0.1% ethanol) for 24 h, loaded with fluo-8 dye and stimulated with carbachol (CCh, 10 uM) or histamine (1 uM) to determine cytosolic elevation of Ca^2+^ ([Ca^2+^]_i_). SA has little effect on **a**-**c**) histamine or **b**-**d**) carbachol-induced [Ca^2+^]_i._ TDI has little effect on **e**-**g**) histamine or **f**-**h**) carbachol-induced [Ca^2+^]_i._ (Data representative of *n* = 3 donors. A, B, E, F: baseline-corrected mean fluorescence in relative fluorescence units (RFU); C, D, G, H: mean ± SEM of n = 3 donors, Area Under the Curve (AUC) of the Ca^2+^ transient; t (s): time in seconds)

In HASM cells, agonist-induced elevation of cytosolic Ca^2+^ ([Ca^2+^]_i_) is a pivotal step in excitation contraction coupling. Amplified Ca^2+^ mobilization mechanisms are implicated in airway hyperreactivity [33–35]. To determine whether augmented [Ca^2+^]_i_ mediates SA-induced AHR and TDI effects on MLC phosphorylation, HASM cells treated with these toxicants were stimulated with contractile agonists carbachol or histamine and [Ca^2+^]_i_ was determined. SA or TDI pre-treatment has little effect on carbachol or histamine-induced [Ca^2+^]_i_ (Fig. 4). DNCB had little effect on carbachol or histamine-induced Ca^2+^ mobilization (data not shown).

### DNCB induces Nrf-2-dependent anti-oxidant response in HASM cells

**Fig. 5:**
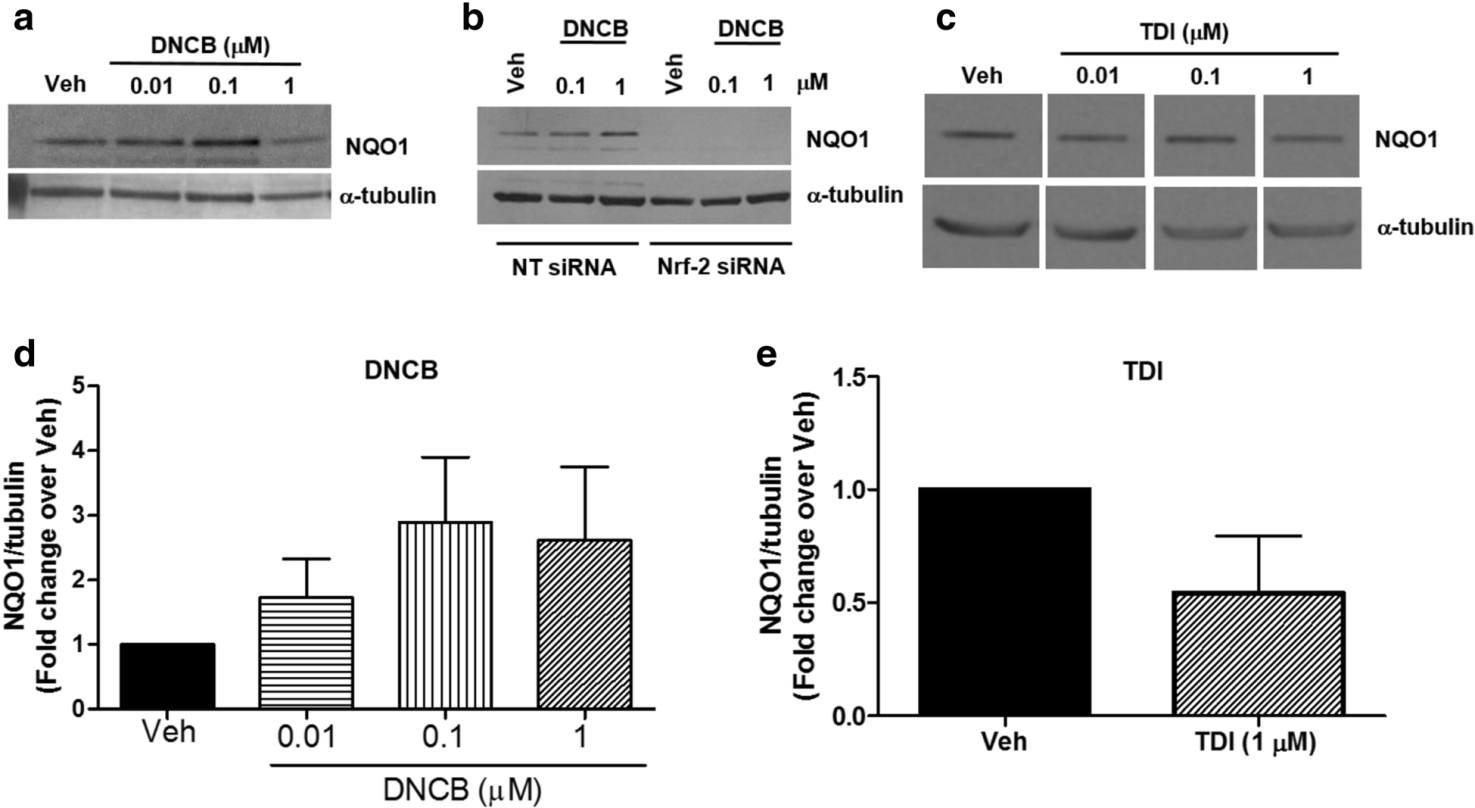
DNCB induces Nrf-2-dependent antioxidant response in HASM cells. HASM cells were exposed to DNCB (0.01, 1, 1 uM) or vehicle (0.1% ethanol) for 24 h and NAD(P) H quinone oxidoreductase (NQO1), a cyto-protective gene product of Nrf-2 induction was determined as a readout for Nrf-2 induction. **a** & **d**) Although statistically not significant due to inter-donor variability (*n* = 5 donors), DNCB increased NQO1 levels in HASM cells. **b**) In HASM cells transfected with Nrf-2-taregted siRNA, DNCB-induced NQO1 induction was inhibited (*n* = 3 donors). **c** & **e**) TDI (0.01, 1, 1 uM), however, has little effect on NQO1 induction in HASM cells (*n* = 3 donors)

Oxidative stress is a common mechanism of injury by many respiratory toxicants. Since, DNCB has little effect on any of the experimental readouts in HASM cells and hPCLS, we tested whether DNCB induces oxidative injury in HASM cells. Nrf-2-mediated antioxidant response, as measured by NQO1 expression, tended to increase in the presence of DNCB (Fig. 5a & d), although there was a higher level of inter-donor variability in this response (*n* = 5 donors). However, siRNA-mediated silencing of Nrf-2, abolished DNCB-induced NQO1 expression in HASM cells, suggesting DNCB induces this antioxidant system in HASM cells (Fig. 5b). TDI, on the other hand, had little effect on NQO1 expression in HASM cells (Fig. 5c & e).

## Discussion

The role of airway smooth muscle cells in AHR and asthma exacerbations is underestimated. We tested the hypothesis that three different toxicants, representing a non-sensitizing irritant (salicylic acid), respiratory sensitizer (toluene diisocyanate) and dermal sensitizer (dinitrochlorobenzene), modulate excitation-contraction coupling to induce AHR. Our findings show that the non-sensitizing irritant SA induces AHR in hPCLS, while the sensitizers TDI and DNCB had little effect. The toxicants had little effect on agonist-induced Ca^2+^ mobilization, while TDI potentiated carbachol-induced MLC phosphorylation in HASM cells. Our findings indicate that, (i) irritants such as salicylic acid may induce AHR independent of inflammatory mediator release, (ii) sensitizers, such as TDI, can act on airway smooth muscle cells to modulate EC coupling, and (iii) the dermal sensitizer DNCB induces Nrf-2 dependent antioxidant response in HASM cells without significant effects on EC coupling or AHR.

Irritant-induced asthma is generally associated with acute inhalation of noxious chemicals in occupational settings [36]. In some individuals, repeated exposure to inhaled irritants may lead to development of long term airway disorders such as reactive airway dysfunction syndrome, characterized by persistent AHR and airway inflammation [37]. SA is a ubiquitous chemical in cosmetic products, with a potential to be inhaled by consumers and cosmetic industry workers. Non-prescription cosmetic products contain ~ 5% (*w*/*v*) of SA, which is equivalent to 362 mM of active compound. Our findings show that a concentration two orders of magnitude less than that of 5% (w/v) SA is sufficient to enhance carbachol-induced bronchoconstriction in hPCLS, without inducing inflammatory mediator release. Well known respiratory irritants, such as chlorine, typically induce airway epithelial injury, inflammatory mediator release and oxidative stress [38].

Enhanced Ca^2+^ mobilization by contractile agonists is one of the mechanisms through which cytokines and certain toxicants mediate airway hyper-reactivity [23, 39]. Our past studies showed that toxicants could also amplify Ca^2+^ sensitization pathways in HASM cells to elicit AHR [1]. While SA induced AHR in PCLS with little effect on MLC phosphorylation, TDI amplified MLC phosphorylation in HASM cells without enhancing bronchoconstriction. These observations suggest that SA-induced AHR potentially originates from actin polymerization in HASM cells [25]. Alternatively, airway epithelial cells may have a prominent role in SA-induced AHR. Airway epithelium provides the first line of physical barrier against inhaled toxicant and in airway diseases like asthma, injured airway epithelium allows these environmental toxicants to reach sub-epithelial tissues [40]. It is likely that airway epithelial cells primarily mediate SA-induced AHR in hPCLS, through paracrine modulation of ASM cells. Future studies will test this hypothesis using in vitro co-culture of HASM and air-liquid interface (ALI)-differentiated epithelial cells.

TDI exposure levels in occupational settings range between 5 and 20 ppb [5]. Bronchial challenge experiments in human subjects found that TDI, at levels as low as 11 ppb, elicit asthmatic response in about 40% of the subjects [41]. Although the highest concentration of TDI (1 μM) in our experiments translate to ~ 170 ppb, this concentration was non-toxic and failed to elicit AHR in hPCLS. The lack of AHR by TDI, despite amplified MLC phosphorylation in HASM cells, supports the theory that SA mediates AHR through actin polymerization. Alternatively, it is plausible that airway epithelial cells in hPCLS have an inhibitory role on TDI-induced airway hyperreactivity. Airway epithelial cells provide the first line of defense against toxicants through their synthetic and physical barrier functions. In hPCLS, the synthetic role of epithelial cells is more relevant than the barrier function. Although Nrf-2-dependent antioxidant response is not induced by TDI in HASM cells, airway epithelial cells may mount an anti-oxidant defense upon TDI exposure to modulate their secretory response to TDI. Studies show that thioredoxin-dependent antioxidant mechanisms are upregulated in airway epithelial cells in response to exposure to pathogens and toxicants to confer protection [42, 43], suggesting similar mechanisms may be at play in hPCLS exposed to TDI. In order to test this hypothesis, future studies should directly measure cell shortening in HASM cells co-cultured with ALI-differentiated epithelial cells and treated with TDI [35, 44].

Clinically relevant DNCB doses have been reported from dermal sensitization studies in humans. Dermal challenge studies showed that DNCB levels ranging between 0.8–3.6 μg.cm^− 2^ (~ 4–18 μM) were capable of eliciting a skin reaction depending on the sensitizing DNCB dose [45]. The lack of AHR or inflammatory mediator release by sensitizing toxicant DNCB in hPCLS confirms earlier findings that circulating innate and adaptive immune cells may be necessary to mediate the effects of this toxicant. Since they are low molecular weight (LMW) antigens, TDI and DNCB need to form adducts with other proteins to become immunologically reactive antigens (“haptenized antigens”) [46, 47]. It is likely that TDI or DNCB form such adducts with proteins in airway structural cells, to directly modulate the functions of those proteins. Amplified agonist-induced MLC phosphorylation by TDI in HASM cells may be the result of this non-specific adduct formation.

We previously reported that Nrf-2-dependent cyto-protective response had little effect on EC coupling in HASM cells [1]. Nrf2-dependent antioxidant response to DNCB is potentially beneficial since it counters ROS-mediated injury. DNCB induces Nrf2-dependent cyto-protective response in a variety of cell types [48]. Studies showed that DNCB-induced Nrf2 activity attenuated apoptosis in dendritic cells by modulating BCL2 expression [49]. It is likely that Nrf2 induction plays a similar role in HASM cells.

These toxicants, particularly the SA, may also act through neurogenic mechanisms to induce AHR. Our in vitro model has limitations to study neurogenic mechanisms. Similarly, lack of circulating inflammatory and immune cells in hPCLS restricts our interpretations to mechanisms in the airway structural cells.

## Conclusion

In summary, our findings show that the non-sensitizing toxicant salicylic acid can evoke AHR in hPCLS, manifested as a leftward shift in the concentration response curve to carbachol. This occurs in the absence of inflammatory mediator release. The sensitizers TDI and DNCB, while modulating HASM cell signaling related to EC coupling and oxidative injury, failed to induce AHR in hPCLS. We can conclude that the investigated toxicants modulate airway structural cells, with a potential to induce AHR in susceptible individuals with airway diseases, such as asthma and COPD.

## Additional files

Additional file 1: **Table S1**. List of cytokines/chemokines screened by Luminex Multi-analyte array (DOCX 293 kb)

Additional file 2: **Figure S1.** DNCB has little effect on carbachol-induced bronchoconstriction in hPCLS. Precision-cut human lung slices (hPCLS) from normal human lung donors were exposed DNCB (0.1–10 uM, vehicle 0.1% ethanol) for 24 h, followed by a carbachol (CCh) concentration-response for bronchoconstriction. A-D) DNCB has little significant effect on carbachol-induced bronchoconstriction in hPCLS (*n* = 3–8 donors) (PDF 41 kb)

## Abbreviations

[Ca^2+^]_i_: intracellular Ca^2+^ level
AHR: airway hyperresponsiveness
ALI: Air-Liquid Interface
ASM: airway smooth muscle
AUC: Area Under the Curve
BCA: Bicinchoninic Acid
CaMKII: Ca^2+^-calmodulin kinase
CCh: Carbachol
COPD: Chronic Obstructive Pulmonary Disease
DNCB: 1-chloro-2,4- dinitrobenzene
EC: Excitation-Contraction
E_max_: maximal response
FBS: Fetal Bovine Serum
FEV_1_: Forced Expiratory Volume in 1 s
HASM: Human Airway Smooth Muscle
hPCLS: human precision-cut lung slices
IL: Interleukin
LDH: Lactate Dehydrogenase
logEC_50_: concentration of half-maximal response
MLC: Myosin Light Chain
NQO1: NAD(P) H Quinone Oxidoreductase 1
Nrf-2: Nuclear factor erythroid 2(NFE2)-related factor 2
pMLC: phosphorylated MLC
RFU: Relative Fluorescence Units
ROS: Reactive oxygen species
SA: Salicylic acid
TDI: Toluene diisocyanate
TNF-α: Tumor Necrosis Factor - α
