# Supplementary material for: Salicylic acid amplifies Carbachol-induced bronchoconstriction in human precision-cut lung slices
Source: Respir Res. 2019 Apr 11;20:72. doi: 10.1186/s12931-019-1034-x (PMC6458705; doi:10.1186/s12931-019-1034-x)

**Supplementary Tables**

**Table S1. List of cytokines/chemokines screened by Luminex Multi-analyte array**


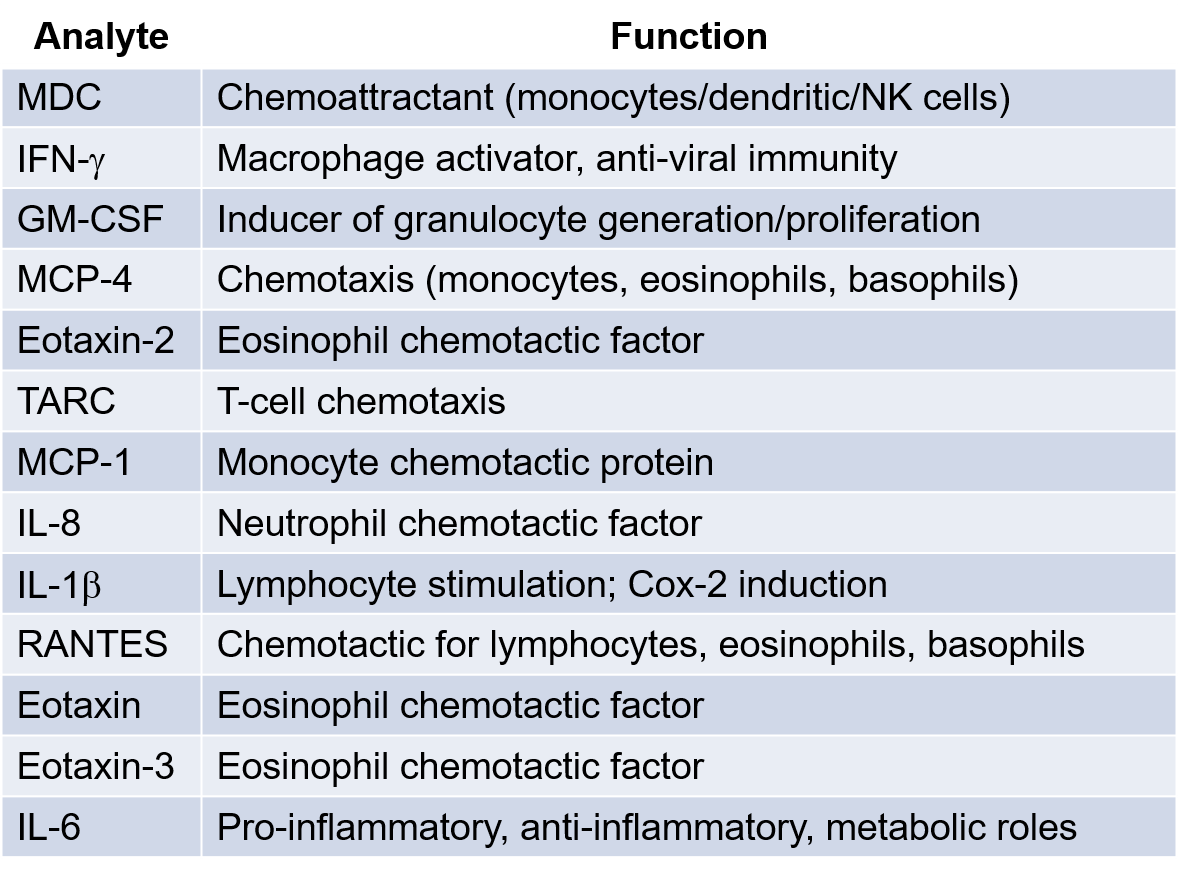

Supplement: Supplementary file 1 — Table S1. List of cytokines/chemokines screened by Luminex Multi-analyte array (DOCX 293 kb) [file 12931_2019_1034_MOESM1_ESM.docx]
