# Supplementary figures and images for: Salicylic acid amplifies Carbachol-induced bronchoconstriction in human precision-cut lung slices
Source: Respir Res. 2019 Apr 11;20:72. doi: 10.1186/s12931-019-1034-x (PMC6458705; doi:10.1186/s12931-019-1034-x)

Figure S1. DNCB has little effect on carbachol-induced bronchoconstriction in hPCLS

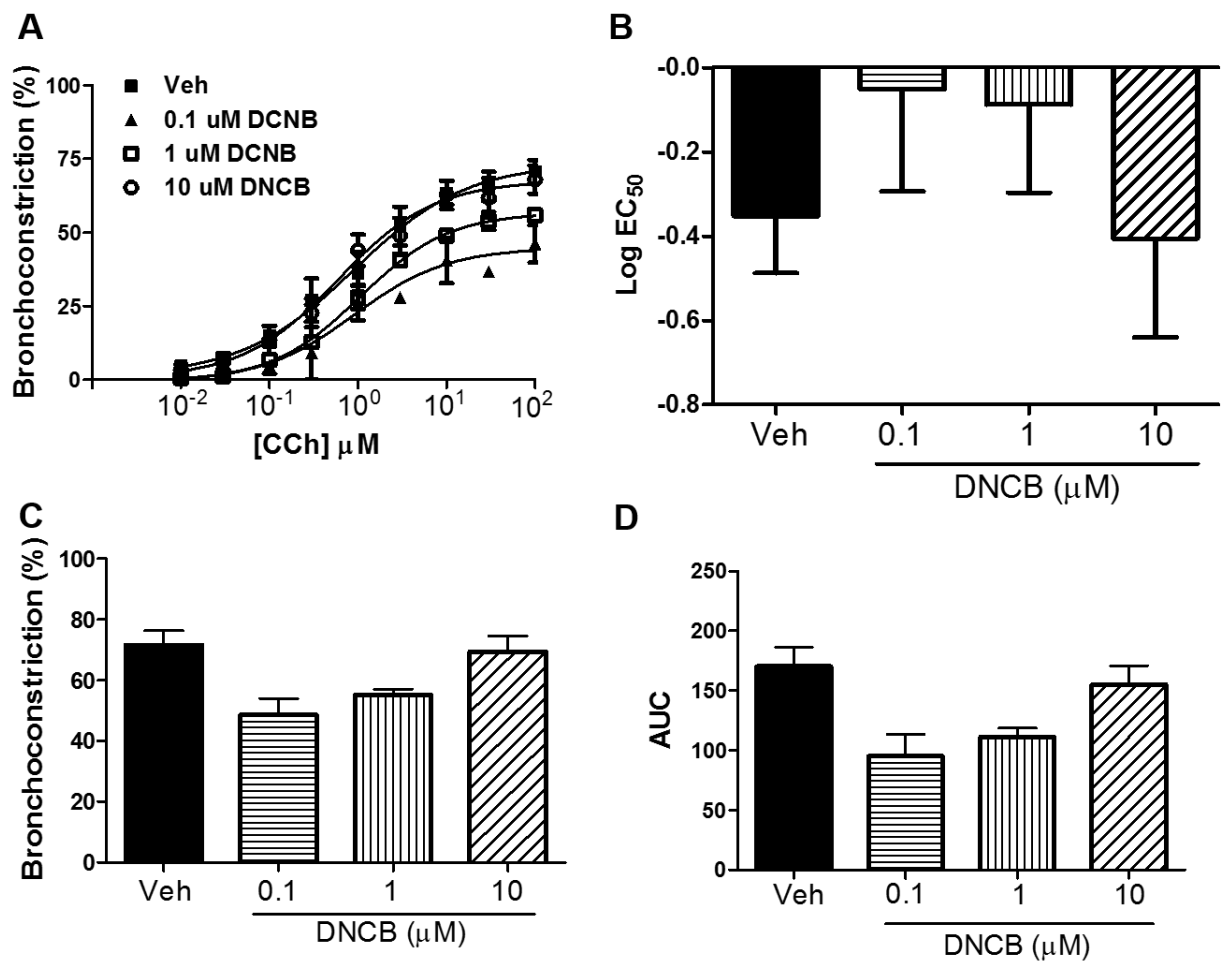

Supplement: Supplementary file 2 — Figure S1. DNCB has little effect on carbachol-induced bronchoconstriction in hPCLS. Precision-cut human lung slices (hPCLS) from normal human lung donors were exposed DNCB (0.1–10 uM, vehicle 0.1% ethanol) for 24 h, followed by a carbachol (CCh) concentration-response for bronchoconstriction. A-D) DNCB has little significant effect on carbachol-induced bronchoconstriction in hPCLS (n = 3–8 donors) (PDF 41 kb) [file 12931_2019_1034_MOESM2_ESM.pdf]
